# Supplementary material for: Identifying suitable reference genes for gene expression analysis in developing skeletal muscle in pigs
Source: PeerJ. 2016 Dec 13;4:e2428. doi: 10.7717/peerj.2428 (PMC5157201; doi:10.7717/peerj.2428)
Supplement: Supplemental Information 1 [file peerj-04-2428-s001.pdf]

| | API5 | AP1S1 | B2M | DRAP1 | GAPDH | H3F3 | PPIA | RHOA |
|---|---|---|---|---|---|---|---|---|
| E33 | 24.15867 | 25.06267 | 22.443 | 24.581 | 17.59467 | 18.33267 | 18.776 | 19.685 |
| E40 | 25.17333 | 25.92567 | 22.048 | 24.67567 | 18.01467 | 19.04 | 19.47133 | 20.34233 |
| E45 | 24.70733 | 26.013 | 21.47167 | 23.956 | 17.80033 | 18.443 | 19.60333 | 20.12467 |
| E50 | 24.02433 | 25.21133 | 21.21833 | 23.61667 | 16.804 | 17.98567 | 18.83267 | 19.183 |
| E55 | 24.28233 | 25.25367 | 21.74567 | 24.788 | 16.72167 | 18.24467 | 18.95433 | 19.24533 |
| E60 | 23.55267 | 24.22 | 21.16167 | 23.9605 | 16.35467 | 17.67367 | 18.77633 | 18.79733 |
| E65 | 24.17133 | 25.22333 | 22.17233 | 24.97867 | 16.678 | 18.09833 | 19.26967 | 19.31733 |
| E70 | 24.87467 | 25.38167 | 22.44067 | 24.88933 | 17.135 | 18.26767 | 20.069 | 19.556 |
| E75 | 25.69133 | 26.19633 | 23.27867 | 25.14433 | 17.182 | 19.77733 | 21.01533 | 20.44367 |
| E80 | 25.23367 | 26.01267 | 22.38067 | 24.82533 | 17.144 | 18.96467 | 20.034 | 19.66067 |
| E85 | 25.27567 | 26.06067 | 22.966 | 26.02067 | 17.11267 | 19.25033 | 20.86633 | 20.219 |
| E90 | 25.851 | 26.65067 | 23.92367 | 28.25933 | 17.126 | 19.58933 | 21.21867 | 20.34733 |
| E95 | 26.30333 | 27.97333 | 24.90033 | 28.154 | 17.553 | 20.66133 | 22.32833 | 21.59733 |
| E100 | 25.8295 | 27.31567 | 23.79067 | 28.645 | 17.39667 | 20.41033 | 21.897 | 20.888 |
| E105 | 24.988 | 26.88933 | 21.62767 | 27.40367 | 16.54267 | 18.49133 | 20.511 | 20.04767 |
| D0 | 23.81333 | 25.561 | 21.84467 | 26.102 | 15.589 | 18.33933 | 19.057 | 19.39667 |
| D10 | 24.829 | 26.35867 | 20.10167 | 24.80467 | 14.50633 | 18.70667 | 19.19833 | 19.924 |
| D20 | 25.155 | 26.717 | 20.28567 | 26.58267 | 14.48267 | 18.875 | 20.84133 | 20.72667 |
| D30 | 25.31567 | 27.33133 | 20.01467 | 26.53333 | 14.33567 | 18.89433 | 21.134 | 20.97333 |
| D40 | 24.49133 | 26.32067 | 18.695 | 24.63667 | 14.089 | 18.545 | 20.024 | 20.24567 |
| D60 | 24.91967 | 26.908 | 20.04433 | 24.79567 | 14.315 | 18.96667 | 19.872 | 20.36167 |
| D80 | 25.22533 | 27.741 | 20.199 | 27.35367 | 14.30033 | 19.23933 | 21.65733 | 20.62133 |
| D100 | 25.30667 | 27.26467 | 20.287 | 27.037 | 14.31733 | 19.23733 | 20.876 | 20.303 |
| D140 | 25.25633 | 27.258 | 21.872 | 26.381 | 14.34267 | 19.499 | 21.32633 | 20.572 |
| D160 | 25.137 | 27.80733 | 20.989 | 26.79967 | 14.392 | 19.12433 | 21.48533 | 20.52433 |
| D180 | 24.53933 | 27.14167 | 20.921 | 24.93767 | 14.119 | 19.43567 | 20.81933 | 20.53267 |

| RNF7 | RPL32 | RPS18 | TBP | WSB | VAPB | ACTB |
|---|---|---|---|---|---|---|
| 23.328 | 19.38367 | 17.53633 | 23.44867 | 23.274 | 22.10667 | 17.55667 |
| 23.574 | 19.97367 | 18.15833 | 25.03967 | 23.457 | 22.54667 | 18.85667 |
| 23.32833 | 19.524 | 17.99733 | 24.53467 | 23.90833 | 22.29 | 18.43667 |
| 22.12967 | 18.672 | 17.54633 | 23.67533 | 22.89533 | 22 | 17.44 |
| 22.04967 | 19.08367 | 17.68667 | 23.926 | 23.278 | 22.34333 | 17.79667 |
| 21.75767 | 18.66233 | 17.41167 | 23.87833 | 22.539 | 21.75 | 17.29 |
| 21.771 | 19.29533 | 17.84033 | 23.97133 | 23.14467 | 22.32 | 16.99333 |
| 22.54033 | 19.90733 | 18.09067 | 24.59167 | 24.16733 | 22.79 | 17.66333 |
| 23.79867 | 20.94633 | 18.81033 | 25.008 | 24.864 | 23.4 | 18.32667 |
| 22.85767 | 20.23467 | 18.46 | 24.45567 | 23.94033 | 22.71667 | 18.30333 |
| 23.82467 | 21.00133 | 18.99533 | 24.671 | 24.42333 | 23.12333 | 18.98667 |
| 23.92267 | 21.456 | 19.111 | 25.388 | 25.238 | 23.39667 | 19.10333 |
| 24.97533 | 23.19733 | 19.757 | 25.16533 | 25.45333 | 23.88333 | 18.88667 |
| 24.56367 | 22.251 | 19.49967 | 25.07867 | 24.83667 | 23.59333 | 18.47 |
| 22.67433 | 20.58233 | 18.65233 | 24.313 | 24.07467 | 22 | 18.42 |
| 22.30867 | 19.582 | 17.49033 | 23.97333 | 22.466 | 20.68667 | 18.59333 |
| 21.77467 | 20.65867 | 19.241 | 24.91567 | 23.12633 | 22.02667 | 17.39667 |
| 22.747 | 20.896 | 19.31433 | 25.34233 | 23.791 | 22.96 | 18.33 |
| 22.50033 | 21.29067 | 19.58967 | 26.14333 | 23.54933 | 22.19 | 19.28667 |
| 22.42167 | 20.319 | 18.87667 | 24.66267 | 23.048 | 22.89667 | 18.13333 |
| 22.14333 | 20.693 | 19.22233 | 24.66867 | 23.10467 | 21.77 | 17.75667 |
| 23.39267 | 21.447 | 19.268 | 25.28533 | 23.76033 | 21.98667 | 20.34333 |
| 21.90567 | 21.37067 | 19.663 | 25.698 | 22.71967 | 21.73333 | 18.32333 |
| 22.21467 | 21.818 | 19.40333 | 26.31033 | 22.79033 | 21.78333 | 19.77667 |
| 22.40667 | 21.963 | 19.66733 | 25.48133 | 22.91033 | 21.80333 | 20.96667 |
| 22.48067 | 20.84367 | 19.28767 | 25.301 | 22.326 | 21.26 | 18.73667 |
